# A review and critical appraisal of central axis flaps in axillary and elbow contractures

**DOI:** 10.1186/s41038-017-0079-7

**Published:** 2017-05-04

**Authors:** Durga Karki, Rajeev B. Ahuja

**Affiliations:** 1 Department of Burns, Plastic and Maxillofacial Surgery, VM Medical College and Safdarjung Hospital, New Delhi, 110029 India; 2 Department of Burns and Plastic Surgery, Lok Nayak Hospital and associated Maulana Azad Medical College, New Delhi, 110 002 India

**Keywords:** Central axis flap, Subcutaneous pedicled flap, Propeller flap, Axillary contracture, Elbow contracture

## Abstract

Contractures of the axilla and elbow can produce a significant impact on quality of life by reducing the ability to perform activities of daily living. Varieties of techniques are available for resurfacing defects following contracture release but graft or flap loss, donor-site morbidity, esthetics, and recurrences are still challenges for reconstructive surgeons. Central axis “propeller” flaps based on a random, subcutaneous pedicle were first described for axillary and elbow contractures to deploy the unburnt skin of axillary dome in type I and II contractures (Kurtzman and Stern) by moving them 90° to straddle the contracting bands. This strategy provided better esthetics and avoided prolonged splinting. Over more than two decades, there have been several design modifications of these flaps with extended applications to cubital fossa. A comprehensive review of published literature on the topic is presented to discuss classifications, design modifications, and applications of such flaps in managing axillary and elbow contractures.

## Background

Contractures of axilla and elbow are one of the most common functionally limiting sequelae of burn injury as they profoundly affect the hand function by influencing the strategic positioning of the hand [1]. Therefore, these contractures can produce a significant impact on quality of life by reducing the ability to perform activities of daily living. A variety of techniques are available for release of contractures, including simple skin grafting, local flaps [2–7], regional flaps [8–16], central axis flaps [17–25], perforator flaps [26–29], and free-tissue transfers [30, 31]. However, graft or flap loss, donor-site morbidity, esthetics, and recurrences are still challenges for reconstructive surgeons.

Propeller flaps are an important addition to the armamentarium for management of soft tissue defects. The concept of propeller flap has evolved over the past few decades with increasing knowledge of the location and vascular territory perfused by perforators. Since central axis propeller flaps are eminently suitable in many cases of axillary and elbow contractures, this review discusses the definition, classification, design modifications, and indications of such flaps primarily for these applications.

## Review

### Definitions and concept

As a subcutaneous pedicle is under the center of every flap, these methods are categorized as “central axis flaps.”

The term “Propeller” was introduced in 1991 by Hyakusoku et al. for reconstruction of contracture in cubital and axillary region [17]. The central axis flap was introduced because of the observation that even extensively burned patients sometimes have healthy skin in axillary or cubital fossa because of joint flexion at the time of injury. The idea was to design flaps in the center of the axillary or cubital fossa, utilizing this unburnt skin, by elevating it as an island on a subcutaneous pedicle. The flap is designed and elevated as a “propeller,” and then rotated by 90° to straddle the area under the contracting bands after contracture release. The remaining donor site is covered with skin grafts. Since then, various modifications of the propeller flap design have been described [19–25].

The concept of “propeller flap” has been refined and modified over the past few years leading to the evolution of “perforator (based) propeller flap” by Hallock [32]. The flap described by Hallock was similar in shape to the one described by Hyakusoku but was based on a skeletonized perforating vessel and was rotated 180°. In the same year, Teo [33] elaborated on the surgical technique and extended the applications of the perforator propeller flaps. The use of such flaps is now popular and features a highly reliable reconstructive method for soft tissue defects in different areas of the body [32–36, 37, 38].

The first meeting on perforator and propeller flaps was conducted in 2009 in Tokyo. The terminology consensus for propeller flaps accepted at this meeting was analogous to the “Gent” consensus on perforator flap terminology [39, 40].

A propeller flap can be defined as an island flap that reaches the recipient site through an axial rotation [40]. Every skin flap can become a propeller flap, but an island flap that reaches the recipient site through an advancement movement or those that move through a rotation and are not completely islanded are excluded from this definition.

### Classification of propeller flaps

Propeller flaps are classified according to Tokyo consensus in 2009 [40] (Table 1).

**Table 1:** Classification of propeller flaps

Type of flap	Pedicle	Definition
Subcutaneous pedicled propeller flap	Random subcutaneous	The flap is based on a random subcutaneous pedicle. The perforators included in pedicle are not visualized or isolated. The flap rotates by 90°.
Perforator pedicled propeller flap	Skeletonized perforating vessel included	Flap is based on a perforator that decides the position of the skin island and is centered over it. The perforator is then skeletonized and freed from the fascial adhesions. The flap rotates between 90° and 180°. This type of flap is most commonly used in reconstructive surgery.
Supercharged propeller flap	Skeletonized perforating vessel with additional vein or artery, or both	If a long pedicled flap is required and the isolated perforator vessel is not providing sufficient arterial inflow or venous outflow, an extra pedicle can be anastomosed. The flap rotates between 90° and 180°.

### Different designs for central axis flaps

#### The “original” propeller flap

**Fig. 1:**
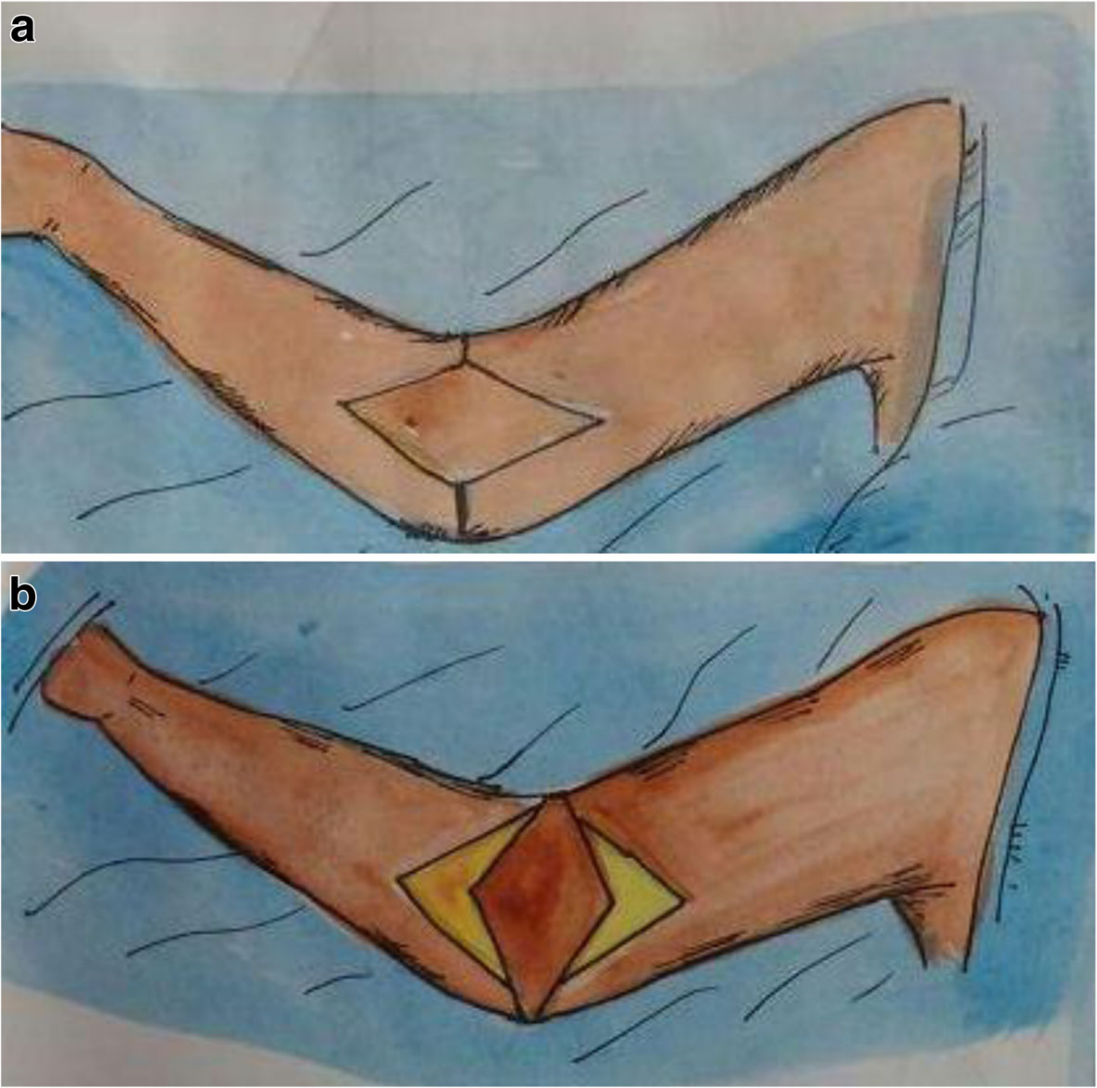
A schematic illustration of the “original” propeller flap design for right elbow contracture. **a** The flap is designed in the center of the cubital fossa along the axis of the arm. **b** After the release of the contracture, the flap is raised and rotated by 90°, like a propeller. The donor area is covered with a skin graft

The original propeller flap method was first introduced by Hyakusoku in 1991 [17]. A diamond-shaped flap is designed along the axis of cubital or axillary fossa depending on the expected size of the defect and the normal skin available. The flap is based on a subcutaneous pedicle only. No attempt is made at Doppler localization of the perforators, preoperatively as the flap is capable of surviving on a broad-based, random, subcutaneous pedicle. The flap is rotated 90° following elevation. The rotation may be either clockwise or anticlockwise, but the flap straddles the horizontal defect created from contracture release. The flap donor area is skin grafted (Fig. 1).

#### Multilobed propeller flap

**Fig. 2:**
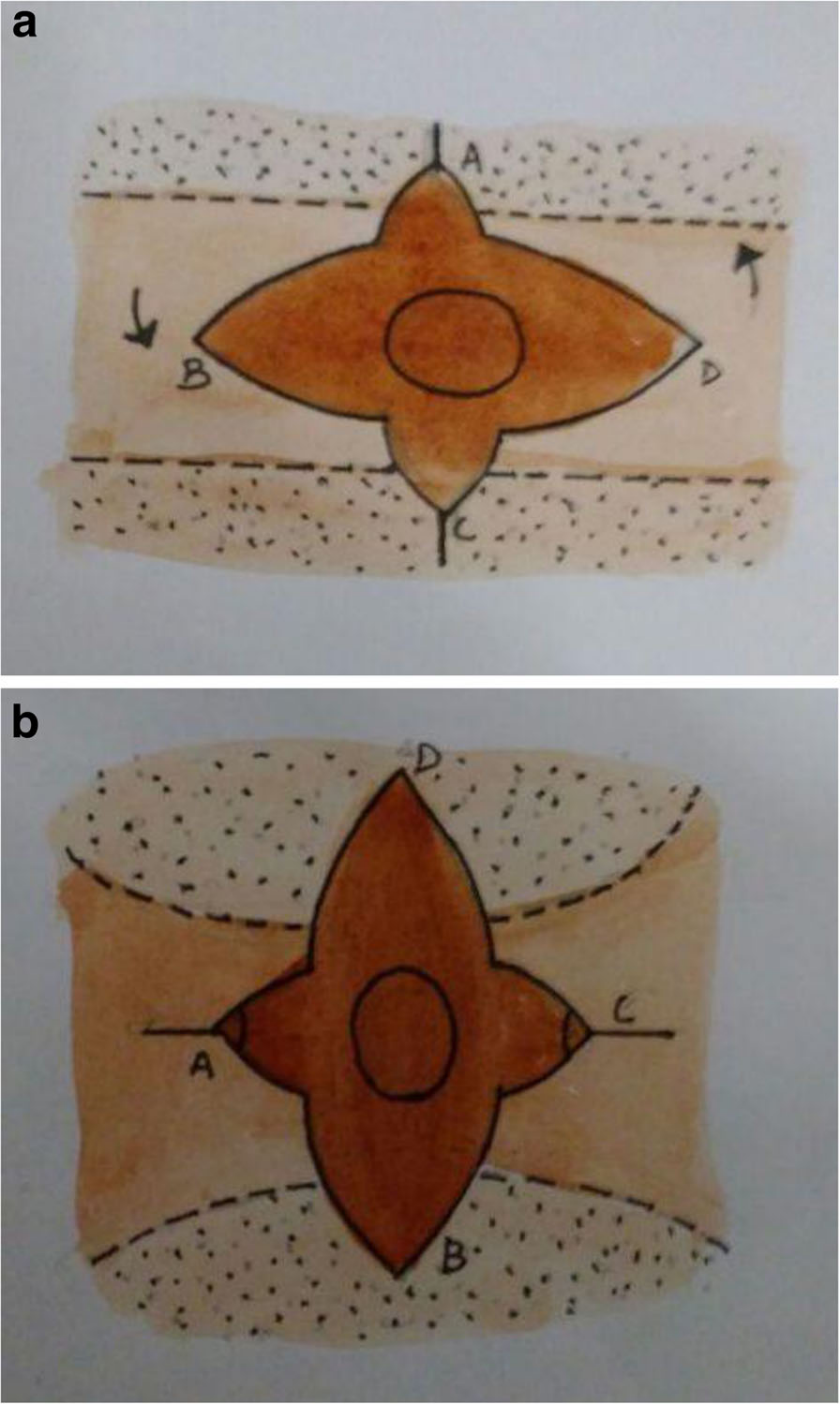
**a**, **b** A schematic illustration of a multilobed propeller flap. The quadrilobed flap, based on a central subcutaneous pedicle is rotated 90° after release of contracture. The donor areas are closed primarily as far as possible

**Fig. 3:**
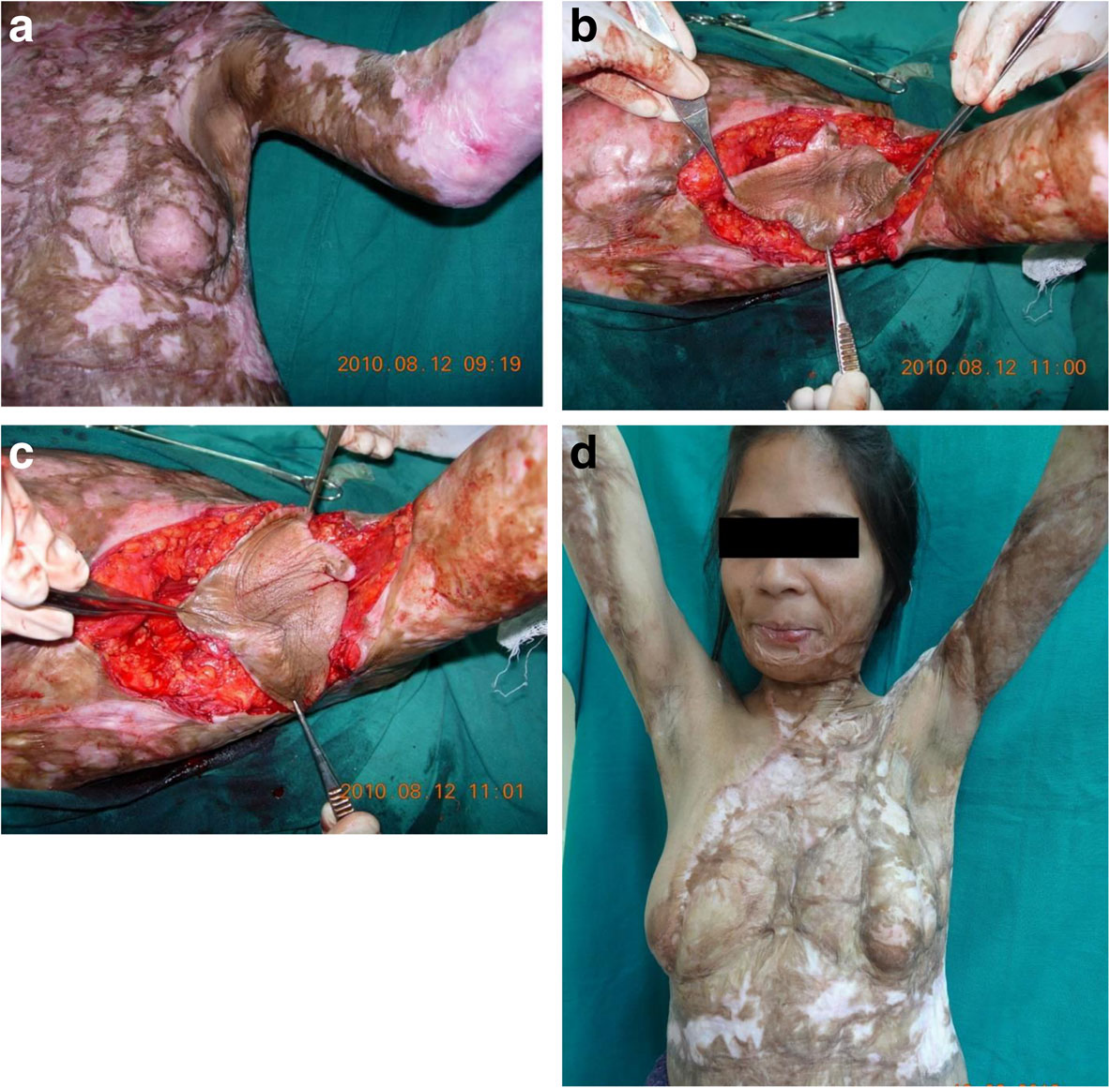
**a** A 32-year-old female with a post-burn, type II, left axillary contracture. The axillary dome is spared but surrounding skin is severely scarred. **b** A quadrilobed flap is raised in the axillary fossa, based on a subcutaneous pedicle. **c** The flap is propelled by 90° to straddle the anterior and posterior axillary contracting bands. The donor area is split skin grafted. **d** An excellent functional and esthetic result at 30 months follow-up. The axillary hairs are well preserved and the flap skin has fully stretched. (Reprinted with the permission from Wolters Kluwer Medknow Publications license no. 4074290918181 [24])

The flap is designed in the form of trilobes or quadrilobes to avoid/minimize the requirement of a skin graft [19]. Flap is similarly based on a thick subcutaneous pedicle and rotated by 90° (Fig. 2). Originally, the lobes were described for surrounding normal skin but these may be designed in scars also. This flap is useful in the release of mild contractures only because the skin is insufficient to resurface a moderate contracture following release. For moderate to severe contractures, it is necessary to graft the donor area after contracture release. Karki et al. reported the use of multilobed propeller flap method in 10 patients with satisfactory results with no flap necrosis or recurrence of contracture [24] (Fig. 3).

#### Pinwheel flap

**Fig. 4:**
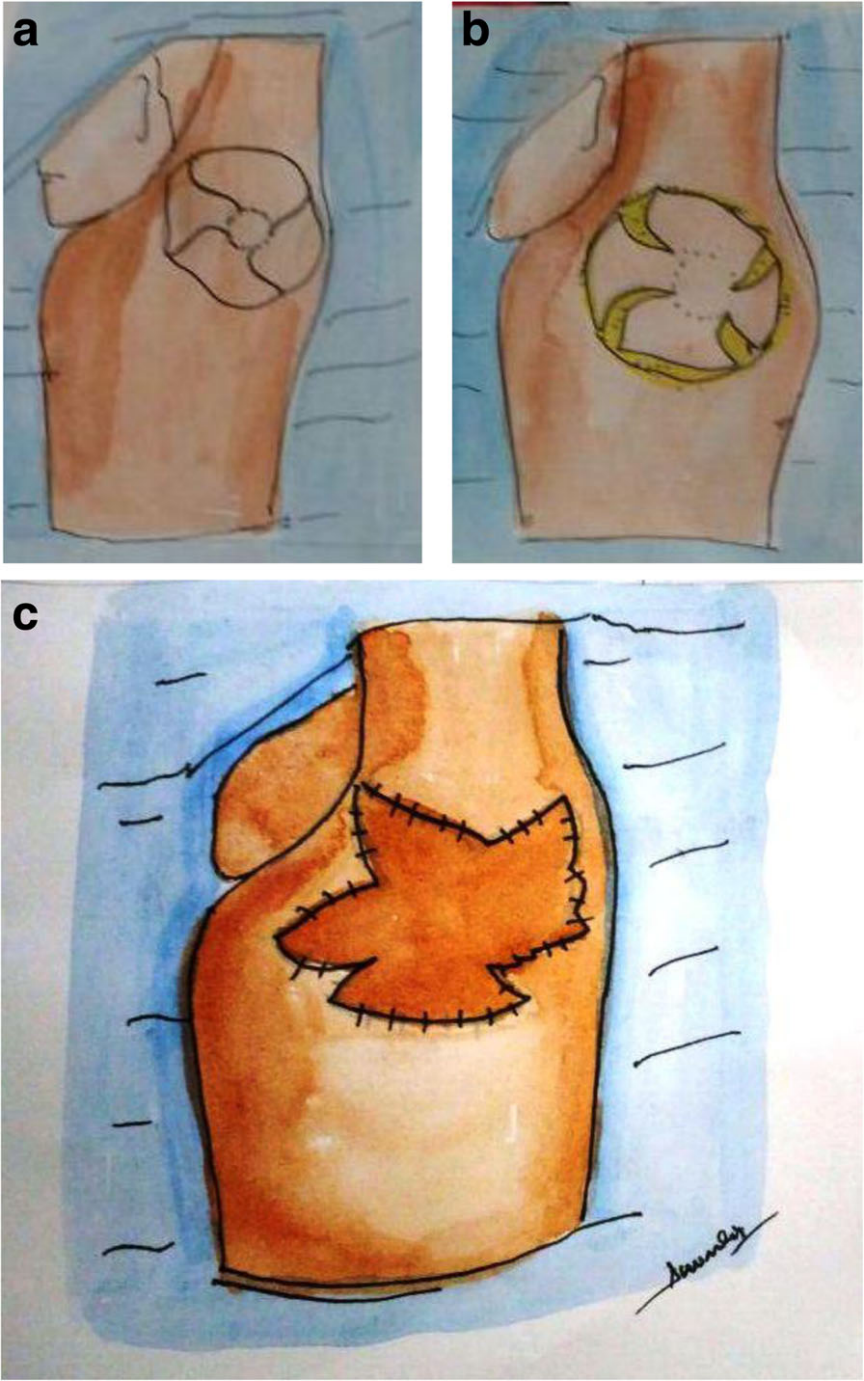
**a**–**c** A schematic illustration of a pinwheel flap design and execution for type II axillary contracture. Flap can be designed with variable number of lobes. Skin flaps spread out like a pinwheel and are rotated into the adjacent defect. Donor areas can usually be closed primarily

To effectively deploy a circular area of unburnt skin, in the axillary or cubital fossa, Hyakusoku et al. in 2006 described a pinwheel flap method [21]. With innovative incisions in the circular piece of skin, flaps can be designed with variable number of lobes to open up like a pinwheel on mobilization. The flaps are based on the central axis only (Fig. 4). It is usually possible to close the donor site primarily. This design of flap also does not release the contracture completely and seems useful for only mild degree of contractures.

#### Scarred propeller flap

**Fig. 5:**
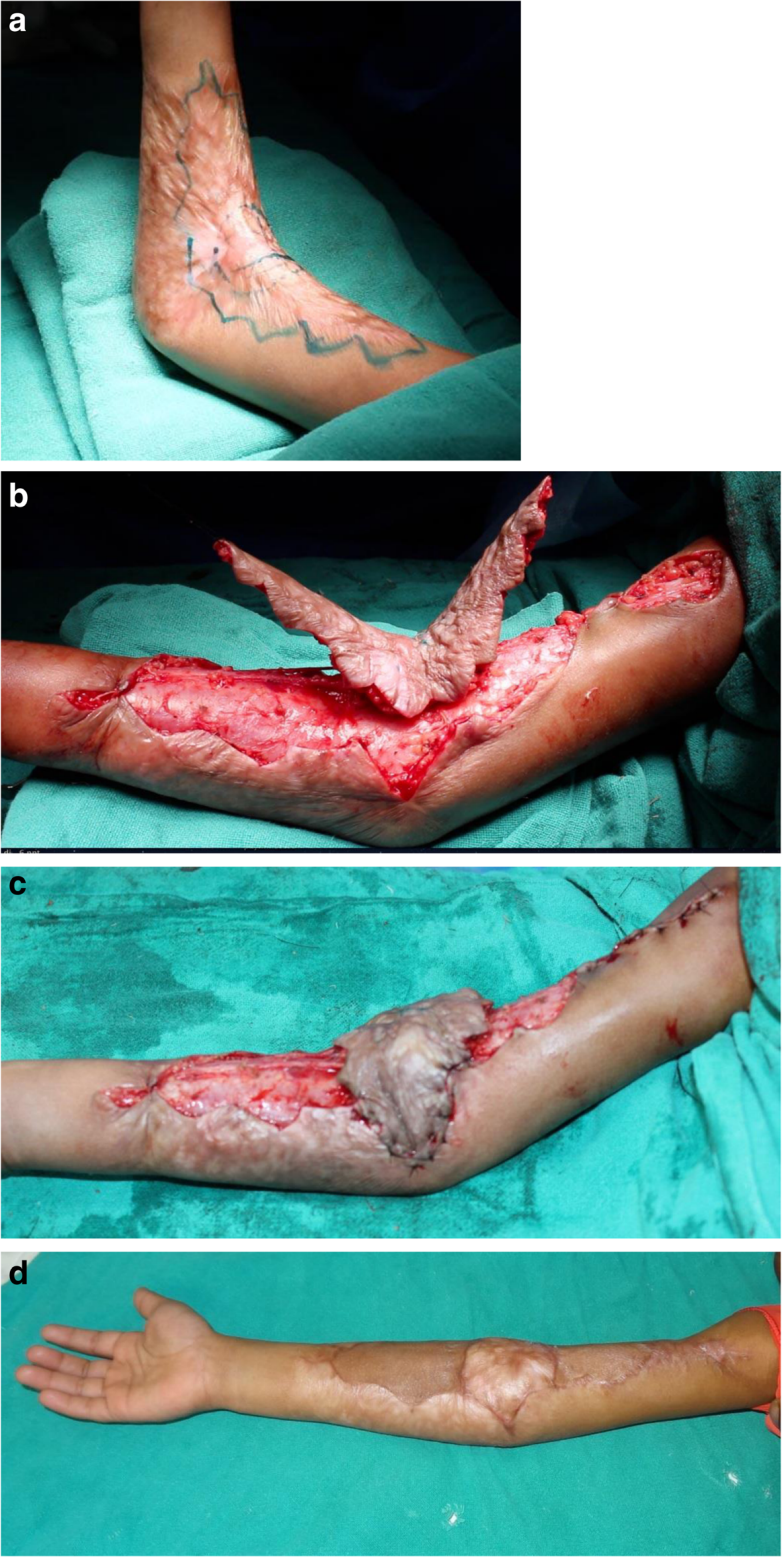
A scarred propeller flap for right elbow contracture. **a** Preoperative flap marking in a 5-year-old child with a 45° elbow contracture. **b** A flap is raised of the central scarred tissue along the axis of the extremity, on a subcutaneous pedicle. Note the zig-zag incisions at the flap margin. **c** The flap is rotated by 90°. A small area of the donor defect is being closed primarily, and the rest is covered with a split skin graft. **d** Full elbow extension at 1 year postoperative follow-up

In mild contractures, a central axis flap may be raised of the contracting band itself [22]. A broad-based flap of the contracting band can be raised on its central axis and rotated 90° (Fig. 5). The donor sites can usually be closed primarily. The main disadvantage of such flaps is a poor cosmetic result because the flap is harvested from scarred skin. Deep burns with immature scar have the possibility of impaired skin perfusion, and we avoid raising a flap in this condition. Sometimes, scarred flaps are bulky and may impair movements of the elbow, so a regular follow-up with pressure garments and physiotherapy is necessary.

#### Eight-limb propeller flap

**Fig. 6:**
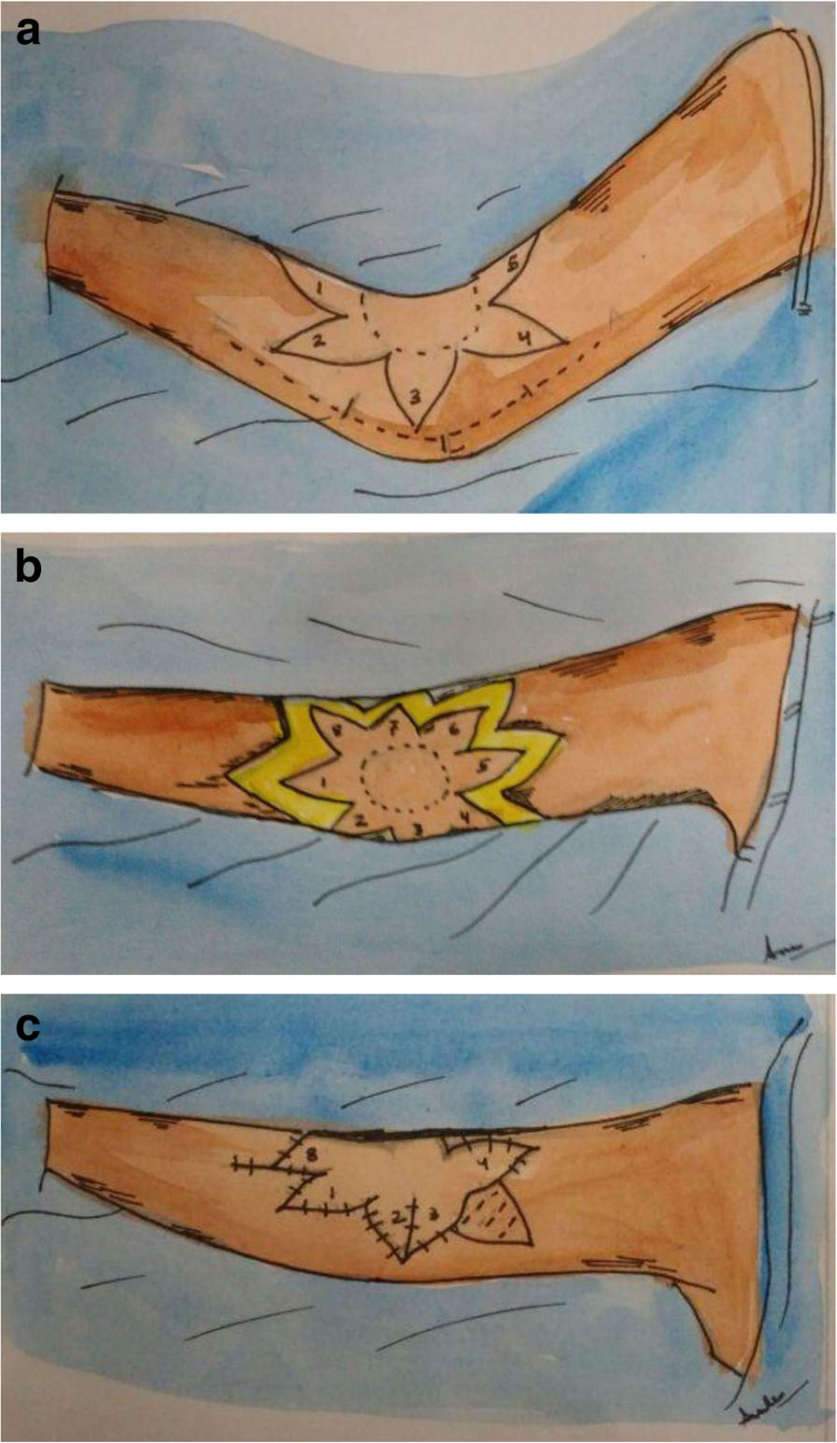
A schematic illustration of an eight-limb propeller flap design for right elbow contracture. **a**, **b** An eight-limb flap marked and elevated with a central axis pedicle. **c** A likely postoperative view with flaps rotated clockwise or anticlockwise. Donor defects closed in a V-Y manner or with a split skin graft

The flap design consists of eight triangular lobes and a central subcutaneous pedicle. The height of each triangle should be equal to the distance between its mid-base and the apex of the adjacent triangle to compensate for the shortening after rotation (Fig. 6). Since the triangular lobes are random flaps, their height should not exceed their base. Each triangular flap is rotated just 45°. It is not necessary to rotate all the triangular flaps in the same direction. In some cases, it may also be possible to partly close primarily the V-shaped donor [23].

#### Zig-zag modified propeller flap

**Fig. 7:**
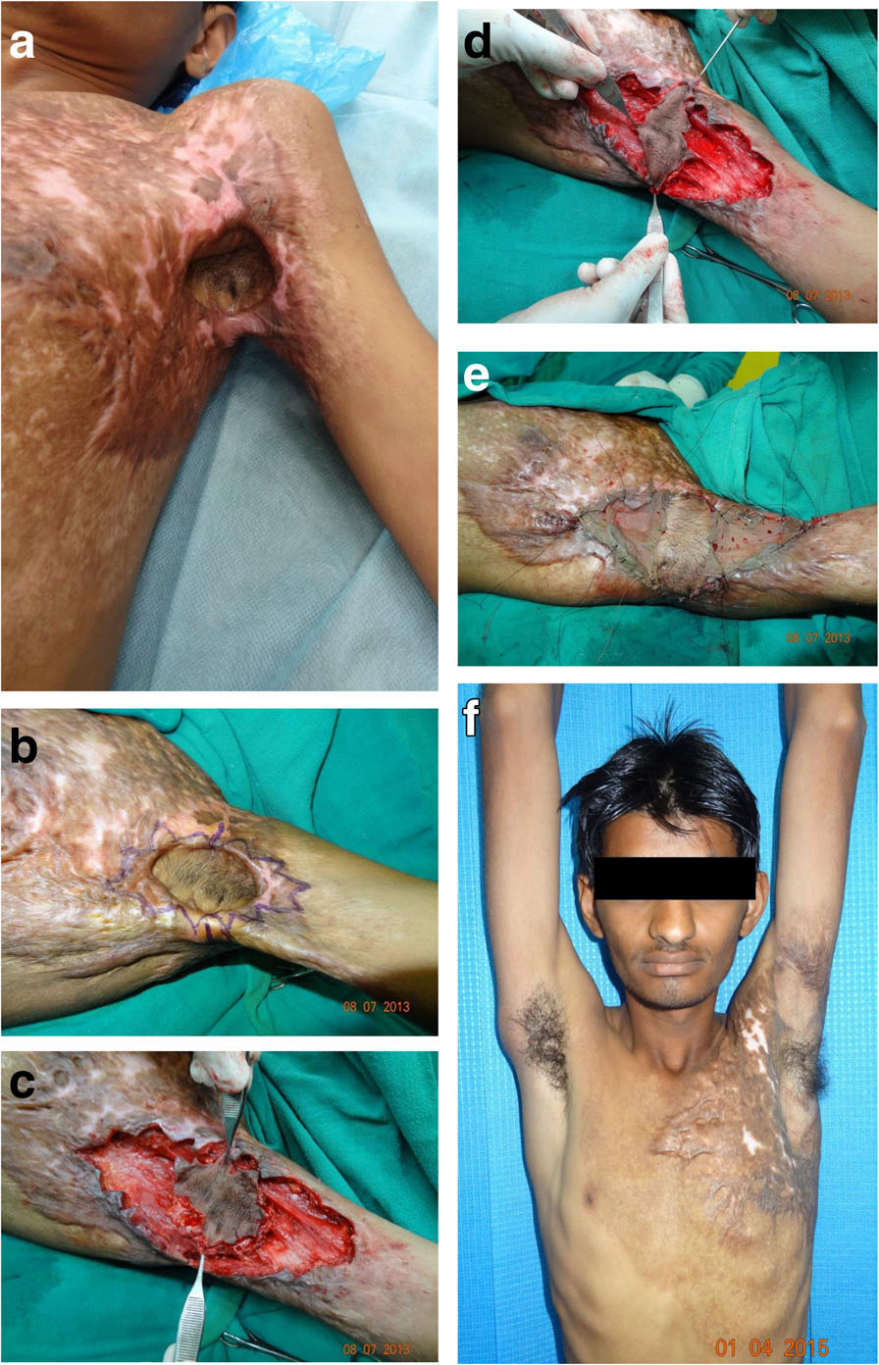
A zig-zag modified propeller flap for type II left axillary contracture in a 29-year-old patient with severe scarring of the anterior chest wall. **a** Preoperative view of with 45° shoulder abduction. **b** A flap marked for central axis pedicle with zig-zag incisions. **c** Release of the axillary contracture and the flap dissected and elevated. **d** The flap has been rotated by 90° to straddle the anterior and posterior contracting bands. **e** Part of the donor area being sutured primarily and the rest covered with a split skin graft. **f** At 3 years follow-up, the patient is showing full shoulder abduction and an excellent esthetic result. **a**, **b**, **e** and **f** were reprinted with permission from *Indian J Plast Surg., 2016* [25]

Karki et al. [25] reported a zig-zag incision around the periphery of the central fossa skin to provide a better esthetic result by interposition of healthy skin in between the skin grafts. This design of subcutaneous pedicled propeller flap is used in type II Kurtzman and Stern axillary [41], and mild to moderate elbow contractures. An elliptical or a diamond shape flap is raised with zig-zag incisions and rotated 90°. It may or may not be possible to primarily close the donor sites depending on the extent of scarring (Fig. 7). Achieving a full range of movement in the joint is more important, and therefore, function should not be compromised to avoid using a skin graft. With a zig-zag flap, there is a possibility of better esthetic result by interpositioning of healthy skin in between the skin grafts which prevents scar band formation and minimizes the recurrence of contracture.

#### “Namaste” propeller flap

**Fig. 8:**
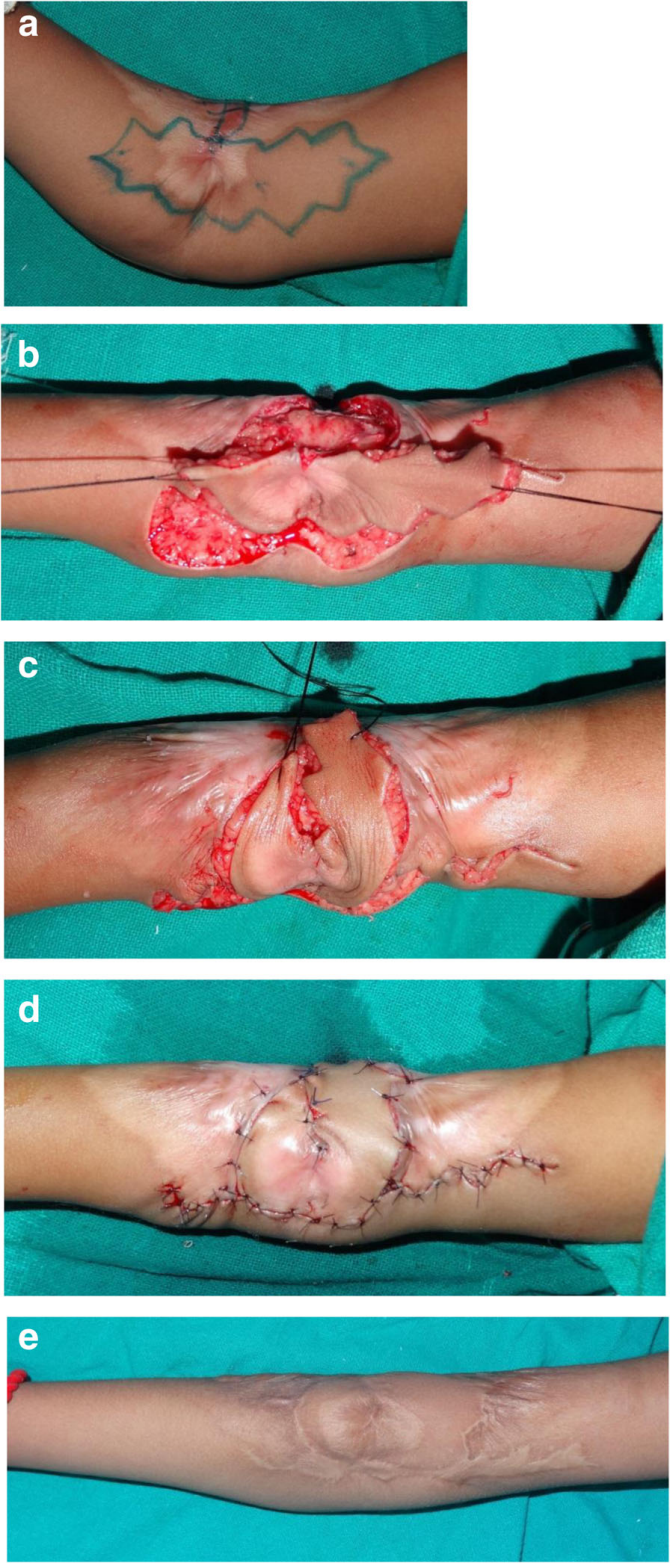
A “Namaste” propeller flap for post-burn contracture of the right elbow. **a** A zig-zag flap outlined for the elbow contracture. **b** The islanded flap is elevated based on a subcutaneous pedicle. **c** Both limbs of the flap are rotated in the same direction by 90°. **d** Flap is inset and the donor sites are closed primarily. **e** A 2-year postoperative results showing full elbow extension

The design of this flap is still unpublished by us (Karki et al.), but it is applicable in axillary contractures involving either anterior or the posterior fold (type 1 contracture) and elbow contracture affecting only medial or lateral side of the cubital fossa. Following release of the contracture, the central axis flap is designed along the long axis of the extremity over residual healthy skin. The width of the flap is equal to half the width of the defect while the length of each limb is 1 cm more than the long axis of the defect. The flaps are raised with a zig-zag incision, and both limbs of the propeller “flap” are rotated by 90° in the same direction. Donor sites are closed primarily. Immediate and complete release of the contracture is possible with complete restoration of range of motion (Fig. 8). The main advantage of this procedure is that the defect created after the release of contracture is completely covered by the flaps. Donor area is primarily sutured, so there is minimal donor-site morbidity.

#### Multiple rhomboid flaps

**Fig. 9:**
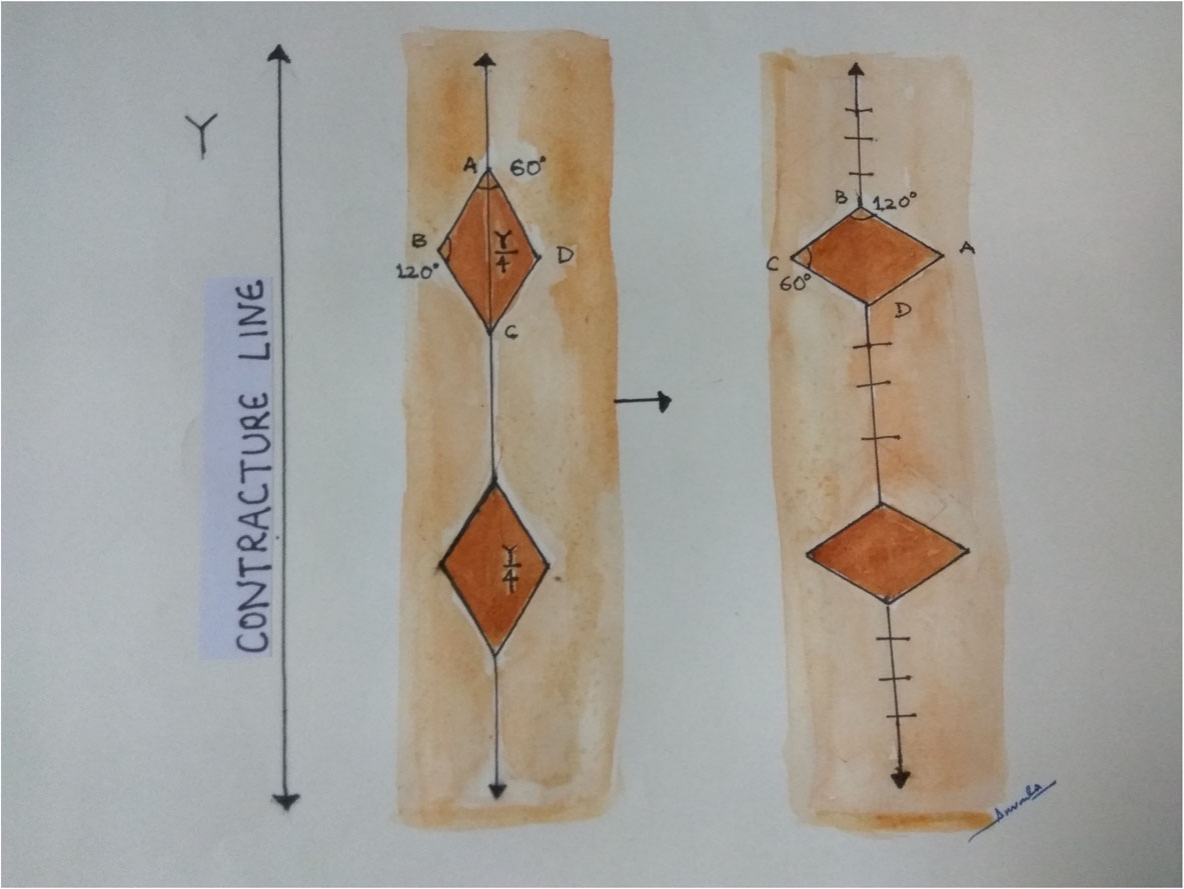
A schematic illustration of planning for multiple rhomboid flaps. Multiple rhomboids with 600 and 1200 angles are designed along the line of contracture. The total length of the flaps should not be less than the half of the contracture length but should also not exceed the total length. It is possible to close the donor defects in V-Y advancement along the long axis

Multiple rhomboid flaps based on subcutaneous pedicles have also been described in reconstruction of post-burn contractures [18]. Rhomboid flaps are designed along the long axis of the contracture band with 1200 flap angles located on the tension lines. The total length of the flaps should not be less than half of the contracture length but should also not exceed the total length. Rhomboid flaps are raised on a broad subcutaneous pedicle without much undermining. Donor defects are closed primarily. Multiple rhomboid flaps are effective in the treatment of long linear and wide contractures with two or more contracture lines (Fig. 9).

## Critical appraisal of central axis flaps

Today, there are multiple options available for reconstruction of post-burn contracture of the axilla and cubital fossa. Traditionally, surgeons have employed Z-plasty [2, 3] or another local flap modification [5–7] to lengthen linear contractures but relied on skin grafting for resurfacing of moderate to severe contractures. Need for prolonged splinting with skin grafting, breadth of scarring in a linear contracture, availability of unburnt skin in the axillary/cubital fossa, and desire for superior esthetics have motivated surgeons to develop innovative local/regional flaps. Good results can be obtained by many techniques. In this regard, the skin graft donor-site morbidity, tolerance for splinting, and complications related to graft or flap failure have dictated individual choices.

The simplest, time-tested reconstructive option has been skin grafting, but the need for prolonged splinting and a potential to recurrence have seen some disenchantment with the procedure. Moreover, skin grafting has limited success in children who are less compliant with postoperative splintage and physiotherapy. However, it seems an ideal choice for resurfacing a type III Kurtzman and Stern [41] axillary contracture or a severe elbow contracture.

Use of local flaps for axillary and elbow contractures has been popular for a longtime. Various local flap options in axilla for type I contracture include random pattern flaps [24], Z-plasty [2, 3], Y-V plasty [4], and a square flap [5], but most of the regional flaps like parascapular flap [12], scapular flap [11], latissimus dorsi flap [8, 9], and thoracodorsal artery perforator (TDAP)-based flap [27–29] are executed in type III contractures, to resurface the dome of the axilla. In 1986, Hyakusoku had also described the use of local fasciocutaneous and musculocutaneous flaps from the surrounding scarred tissue in patients of axilla contracture who did not have unscarred donor sites available [8]. The decision to use scarred skin, as a flap, should be made keeping in mind the depth of the burn scar and previous surgical excisions [22].

However, when the axillary dome is spared, the use of local flaps mentioned above or a local flap rearrangement advanced by Z-plasty, or its likes, can either mar the esthetics of the axilla by sacrificing healthy hairy skin or destroy the potential to employ the unburnt skin in reconstruction. Quite akin are the circumstances with the presence of unburnt skin in the cubital fossa in elbow contractures.

The introduction of the central axis flap, for axillary contractures by Hyakusoku et al., added another option of reconstruction to address type II contractures. The attraction of the technique lies in utilizing the spared axillary fossa skin and propelling it by 90° to straddle the anterior and posterior axillary folds. The flap is based on a subcutaneous pedicle, is extremely simple to raise, and requires no microsurgical skills. Although, the donor area is still covered with skin grafts, it provides excellent functional recovery within 6 months, without splinting. The subcutaneous pedicle is very reliable, and minimal complications have been reported subsequently with this technique.

Perforator-based propeller flaps have overshadowed the application of central axis subcutaneous pedicled propeller flaps. Thoracodorsal artery perforator flap (TDAP flaps) is based on a septocutaneous perforator of circumflex scapular artery. It is increasingly used in axillary contractures to resurface the dome in type III contracture. It provides superior esthetics; less need for prolonged splinting and the donor site can be closed primarily [27–29].

There are several other options in reconstruction besides the central axis flaps, including a square flap [5], a trapeze-flap plasty [6, 7], or a free flap [40, 41]. A square flap has been described for a type I axillary contracture [13, 24]; a trapeze-flap plasty [6, 7] may be applicable in both axillary or elbow contractures; and a free flap [40, 41] is obviously employed in the absence of local tissue and the need to do away with splinting.

Reconstruction of axilla and elbow is difficult in patients with extensive burn because of skin paucity. Various designs for local flaps or central axis flaps offer an option to use any amount of unburnt skin centrally or in the vicinity. Many broad-based or multiple linear contractures can be managed effectively with the use of these flaps, besides these flaps offer better esthetics with superior and stable skin. The central axis flaps also do not dislocate the hair-bearing skin of the axilla. However, because of inherent limitations, many of these flaps are applicable only in mild contractures because if used in moderate contractures, they lead to incomplete release in the first stage. At the same time, these flaps are known to stretch over a period of time improving the functional outcome. There are no defined criteria for dimensions of these flaps that can be safely raised but very long flaps have been elevated and rotated by 90° without significant complications.

## Conclusions

Central axis subcutaneous pedicled flaps are a reliable method of reconstruction in axillary and elbow contractures. They are eminently applicable in mild to moderate contractures but may not permit full contracture release in severe contractures. Better esthetic result, fewer complications, and the possibility to do away with splinting make these flaps a very attractive option.

## References

[1] Huang T, Yang JY, Herndon DN (2012). Management of contractural deformities involving the shoulder (axilla), elbow, hip and knee joints in burned patients. Total burn Care.

[2] Rohrich RJ, Zbar RIS (1999). A simplified algorithm for the use of Z-plasty. Plast Reconstr Surg.

[3] Salam GA, Amin JP (2003). The basic Z-plasty. Am Fam Physician.

[4] Cooper MACS (1990). The multiple Y-V plasty in linear burn scar contracture release. Br J Plast Surg.

[5] Hyakusoku H, Shirai H, Umeda T, Fumiiri M (1985). The use of the square flap method for repair of axillary burn contracture. Plast Reconstr Surg.

[6] Grishkevich VM (1991). The basic types of scar contractures after burns and methods of eliminating them with trapezeplasty flaps. Plast Reconstr Surg.

[7] Grishkevich VM (2010). Trapezoid adipose scar local flap: Postburn lateral truncal contracture elimination with trapeze-flap plasty. J Burn Care Res.

[8] Hyakusoku H, Okubo M, Suenobu J, Fumiiri M. Use of scarred flaps and secondary flaps for reconstructive surgery of extensive burns. Burns. 1986;12:470–4. 10.1016/0305-4179(86)90071-9 3779468

[9] Achauer BM, Spenler CW, Gold ME (1988). Reconstruction of axillary burn contractures with latissimus dorsi fasciocutaneous flap. J Trauma.

[10] Hyakusoku H, Yoshida H, Okubo M, Hirai T, Fumiiri M. Superficial cervical artery skin flaps. Plast Reconstr Surg. 1990;86:33–8. 10.1097/00006534-199007000-00005 2359800

[11] Nisanci M, Er E, Isik S, Sengezer M. Treatment modalities for post-burn axillary contractures and the versatility of the scapular flap. Burns. 2002;28:177–80. 10.1016/s0305-4179(01)00090-0 11900943

[12] Tiwari P, Kalra GS, Bhatnager SK (1990). Fasciocutaneous flaps for burn contractures of the axilla. Burns.

[13] Ogawa R, Hyakusoku H, Murakami M, Koike S (2003). Reconstruction of axillary scar contractures—retrospective study of 124 cases over 25 years. Br J Plas Surg.

[14] Turegan M, Nisanci M, Duman H, Aksu M, Sengezer M (2005). Versatility of the reverse lateral arm flap in the treatment of post-burn antecubital contractures. Burns.

[15] Lewis VL, Cook JQ (1980). The non-delayed thoracoepigastric flap: coverage of an extensive electric burn defect of the upper extremity. Plast Reconstr Surg.

[16] Schwarz RJ (2007). Management of postburn contractures of the upper extremity. J Burn Care Res.

[17] Hyakusoku H, Yamamoto Y, Fumiiri M (1991). The propeller flap method. Br J Plast Surg.

[18] Ertas NM, Kucukcelebi A, Bozdogan N, Celebioglu S (2004). The use of subcutaneous pedicle multiple rhomboid flaps in the treatment of long post burn scar contractures. Burns.

[19] Murakami M, Hyakusoku H, Ogawa R (2005). The multi-lobed propeller flap method. Plast Reconstr Surg.

[20] Murakami M, Hyakusoku H, Ogawa R (2005). The scar band rotation flap. Burns.

[21] Hyakusoku H, Iwakiri I, Murakami M, Ogawa R (2006). Central axis flap methods. Burns.

[22] Aslan G, Tuncali D, Cigsar B, Barutcu AY, Terzioglu A (2006). The propeller flap for postburn elbow contractures. Burns.

[23] Husam H, El-Shaer W (2011). The eight-limb modified propeller flap—a safer new technique. Burns.

[24] Karki D, Mehta N, Narayan RP (2014). Post axillary burn contracture: a therapeutic challenge!. Indian J Plast Surg.

[25] Karki D, Mehta N, Narayan RP (2016). Subcutaneous pedicled propeller flap: an old technique revisited and modified!. Indian J Plast Surg.

[26] El-Khatip H (1997). Island fasciocutaneous flap based on the proximal perforators of the radial artery for resurfacing of burned cubital fossa. Plast Reconstr Surg.

[27] Kim DY, Cho SY, Kim KS, Lee SY, Cho BH. Correction of axillary burn scar contracture with the thoracodorsal perforator-based cutaneous island flap. Ann Plast Surg. 2000;44:181–7. 10.1097/00000637-200044020-00010 10696046

[28] Er EL, Ucar C (2005). Reconstruction of axillary contractures with thoracodorsal perforator island flap. Burns.

[29] Kulahci Y, Sever C, Uygur F, Oksuz S, Sahin C, Duman H (2011). Pre-expanded pedicled thoracodorsal artery perforator flap for post burn axillary contracture reconstruction. Microsurgery.

[30] Ohmori S (1982). Correction of burn deformities using free flap transfer. J Trauma.

[31] De Lorenzi F, Vander Hulst R, Boeckx W (2001). Free flaps in burn reconstruction. Burns.

[32] Hallock GG (2006). The propeller flap version of the adductor muscle perforator flap for coverage of ischial or trochanteric pressure sores. Ann Plast Surg.

[33] Teo TC (2006). Perforator local flaps in lower limb reconstruction. Cir Plast Ibero Latinoam.

[34] Masia J, Moscatiello F, Pons G, Fernandez M, Lopez S, Serret P (2007). Our experience in lower limb reconstruction with perforator flaps. Ann Plast Surg.

[35] Moscatiello F, Masia J, Carrera A, Clavero JA, Larranaga JR, Pons G (2007). The “propeller” distal anteromedial thigh perforator flap: anatomic study and clinical application. J Plast Reconstr Aesthet Surg.

[36] Pignatti M, Pasqualini M, Governa M, Bruti M, Rigotta G (2008). Propeller flaps for leg reconstruction. J Plast Reconstr Aesthet Surg.

[37] Georgescu AV, Matei I, Ardelean F, Capota I (2007). Microsurgical non microvascular flaps in forearm and hand reconstruction. Microsurgery.

[38] Mateev MA, Ogawa R, Trunov L, Moldobaeva N, Hyakusoku H (2009). Shape-modified radial artery perforator flap method: analysis of 112 cases. Plast Reconstr Surg.

[39] Blondeel PN, Van Landuyt K, Monstrey SJ, Hamdi M, Matton GE, Allen RJ, et al. The “Gent” consensus on perforator flap terminology: preliminary definitions. Plast Reconstr Surg. 2003;112:1378–82. 10.1097/01.PRS.0000081071.83805.B6 14504524

[40] Pignatti M, Ogawa R, Hallock GG, Mateev M, Georgescu AV, Balakrishnan G (2011). The “Tokyo” consensus on propeller flaps. Plast Reconstr Surg.

[41] Kurtzman LC, Stern PJ (1990). Upper extremity burn contractures. Hand Clin.

